# Supplementary material for: Probabilistic graphical modelling of early childhood caries development
Source: PLoS One. 2023 Oct 30;18(10):e0293221. doi: 10.1371/journal.pone.0293221 (PMC10615302; doi:10.1371/journal.pone.0293221)
Supplement: S1 File — (DOCX) [file pone.0293221.s001.docx]

Supplementary material 1 – Appendix

Table S1: measured variables in the longitudinal study. In **bold** variables handled to define the eight variables used in the UG modelling and defined in Table S2.

| 4 unit identification variable | Id, Progressive number, Gender, Ethnicity |
| --- | --- |
| 18 general questionnaire variables | Binary variables. If the child   - was born in time, - **has been breastfed, if yes has been breastfed in an exclusive or mixed way,** - **has used the feeding bottle,** - **has used the pacifier,** - has sucked his thumb, - **has received instructions of oral hygiene,** - has suffered tooth or face trauma, - takes fluoride. |
|  | How many times each day the child brushes his teeth: 1, 2 or more then 2? |
|  | For how many months the child has   - **been breastfed,** - **used the feeding bottle,** - **used the pacifier,** - sucked his thumb. |
| 29 variables measured at the three timepoint age 3, 4 and 5 | Binary variables. If the child has   - had tonsils or adenoids surgery, allergies, **good oral hygiene,** only milk teeth, an infantile or normal swallowing, lip competence, normal or oral type of breathing, recurrent otitis, - wears a cross bite, - **drinks juices, tea or sugary drinks**, - **drinks coca cola, fanta or other carbonated drink**s. |
|  | Ternary: if the child   - **eats fruit or vegetables daily, weekly or occasionally,** - **drinks juices, tea or sugary drinks daily, weekly or occasionally,** - **drinks coca cola, fanta or other carbonated drinks daily, weekly or occasionally,** - has mesial, head to head or distal step right occlusion, - has mesial, head to head or distal step left occlusion, - has first, second or third class of right canine key, - has first, second or third class of left canine key, - has a bilateral, right unilateral or left unilateral cross bite, - has a right deflected, centered or left deflected upper midline, - has a right deflected, centered or left deflected lower midline, - has a normal, low or interposed posture of tongue. |
|  | Other variables:   - child age, weight, height, - **number of caries and of fillings,** - mm of overbite and mm of overjet. |

Table S2: variables for the UG modelling constructed following associative and explorative data analysis. The construction of the variables from the original ones in in Table S1 is illustrated in the text. Here, in italics the same variables as in Table S1 and in **bold** a categorization of the original ones. The others are calculated starting from different original variables in Table S1.

| **Variable name** | **Variable type** | **Variable definition** |
| --- | --- | --- |
| Oral hygiene status | Binary | Is the Oral hygiene status of the child adequate or not adequate? |
| Caries variation | Binary | Has the child increased the number of caries at age five, with respect to age three? |
| Breastfeeding type | Ternary | *Which type of breastfeeding has the child received? No breastfeeding, exclusive or mixed (also with feeding bottle) breastfeeding?* |
| Breastfeeding time | Quaternary | **For how many months has the child been breastfed? This is a categorization of the recorded variable.** |
| Use of Pacifier | Quaternary | **For how many months has the child used the pacifier? This is a categorization of the original recorded variable.** |
| Frequency of toothbrushing | Ternary | *How many times per day does the child brush his/her teeth 1, 2 or more than 2?* |
| Consumption of sugared beverages | Ternary | Does the child drink sugary or carbonated sodas 1 time at day, 1 time at week or occasionally? |
| Consumption of vegetables/fruits | Ternary | Does the child eat vegetables 1 time at day, 1 time at week or occasionally? |

Table S3: odds-ratios of caries variation with respect to the other seven selected variables. Significant p-value associated to an odds-ratio highlights a useful variable in order to predict the variation of the number of caries. 95% Confidence intervals for odds-ratios are also calculated.

| **Breastfeeding type** | **odds-ratio** | **left-end confidence interval** | **right-end confidence interval** | **p-value** | |
| --- | --- | --- | --- | --- | --- |
| 1 VS 0 | 2.528 | 0.918 | 8.939 | | 0.101 |
| 2 VS 0 | 3.329 | 1.086 | 12.539 | | 0.048* |
| 2 VS 1 | 1.317 | 0.619 | 2.718 | | 0.463 |
| **Breastfeeding time** |  |  |  | |  |
| 1 VS 0 | 2.395 | 0.791 | 8.937 | | 0.148 |
| 2 VS 0 | 2.437 | 0.832 | 8.929 | | 0.132 |
| 3 VS 0 | 4.469 | 1.360 | 17.608 | | 0.019* |
| 2 VS 1 | 1.018 | 0.462 | 2.269 | | 0.965 |
| 3 VS 1 | 1.866 | 0.732 | 4.703 | | 0.185 |
| 3 VS 2 | 1.833 | 0.740 | 4.446 | | 0.182 |
| **Frequency of toothbrushing** |  |  |  | |  |
| 2 VS 1 | 0.800 | 0.390 | 1.694 | | 0.550 |
| 3 VS 1 | 0.974 | 0.311 | 2.782 | | 0.962 |
| 3 VS 2 | 1.217 | 0.415 | 3.151 | | 0.700 |
| **Oral hygiene status** |  |  |  | |  |
| 2 VS 1 | 673.200 | 126.338 | 12581.880 | | <0.001*** |
| **Consumption of sugared beverages** |  |  |  | |  |
| 2 VS 1 | 0.653 | 0.233 | 1.904 | | 0.419 |
| 3 VS 1 | 270.000 | 47.232 | 5196.620 | | <0.001*** |
| 3 VS 2 | 413.333 | 75.672 | 7796.548 | | <0.001*** |
| **Consumption of vegetables/fruits** |  |  |  | |  |
| 2 VS 1 | 0.868 | 0.445 | 1.704 | | 0.678 |
| 3 VS 1 | 1.283 | 0.267 | 4.774 | | 0.726 |
| 3 VS 2 | 1.478 | 0.310 | 5.413 | | 0.580 |
| **Use of Pacifier** |  |  |  | |  |
| 1 VS 0 | 1.113 | 0.495 | 2.507 | | 0.794 |
| 2 VS 0 | 0.722 | 0.282 | 1.768 | | 0.483 |
| 3 VS 0 | 1.020 | 0.355 | 2.721 | | 0.970 |
| 2 VS 1 | 0.649 | 0.252 | 1.594 | | 0.353 |
| 3 VS 1 | 0.916 | 0.317 | 2.453 | | 0.865 |
| 3 VS 2 | 1.412 | 0.458 | 4.215 | | 0.537 |

Table S4: relative/percentage distribution of joint distribution of Breastfeeding type and time. It highlights structural zeros.

|  |  | | Breastfeeding type | | |
| --- | --- | --- | --- | --- | --- |
|  |  |  | 0 | 1 | 2 |
|  | Breastfeeding time | 0 | 18.38 | 0.00 | 0.00 |
|  |  | 1 | 0.00 | 23.08 | 7.26 |
|  |  | 2 | 0.00 | 25.64 | 10.68 |
|  |  | 3 | 0.00 | 9.40 | 5.56 |

Table S5: F-tests for the estimated UGM. Each row corresponds to a test that tests if a single variable or an interaction between pair of variables can be removed from the estimated UGM without losing information. Significant p-values suggest that the preferable model is the UGM.

|  | Df | Deviance | AIC | F-value | P-value |
| --- | --- | --- | --- | --- | --- |
| UGM | - | 135.82 | 419.57 | - | - |
| Oral hygiene status | 1 | 252.43 | 534.18 | 465 | <0.001*** |
| Consumption of sugared beverages | 2 | 313.52 | 593.27 | 355 | <0.001*** |
| Breastfeeding time | 3 | 177.68 | 455.43 | 56 | <0.001*** |
| Breastfeeding type | 2 | 230.30 | 510.05 | 189 | <0.001*** |
| Use of pacifier | 3 | 137.00 | 414.76 | 2 | 0.194 |
| Caries variation | 1 | 329.82 | 611.58 | 774 | <0.001*** |
| Oral hygiene status - Consumption of sugared beverages | 2 | 287.02 | 566.77 | 302 | <0.001*** |
| Oral hygiene status - Caries variation | 1 | 279.48 | 561.24 | 573 | <0.001*** |
| Oral hygiene status - Breastfeeding time | 3 | 143.22 | 420.98 | 10 | <0.001*** |
| Breastfeeding time - Breastfeeding type | 6 | 361.07 | 632.82 | 150 | <0.001*** |
| Breastfeeding time - Use of pacifier | 9 | 158.62 | 424.37 | 10 | <0.001*** |
