# Supplementary material for: Probabilistic graphical modelling of early childhood caries development
Source: PLoS One. 2023 Oct 30;18(10):e0293221. doi: 10.1371/journal.pone.0293221 (PMC10615302; doi:10.1371/journal.pone.0293221)
Supplement: S2 File — (DOCX) [file pone.0293221.s002.docx]

Supplementary material 2 – Appendix

Table 1_bis: univariate distributions of the eight selected variables used for the UG modelling, considering the total sample of 273 subjects.

| **Caries incidence** | **% (count)** | **Oral hygiene status** | **% (count)** |  |
| --- | --- | --- | --- | --- |
| 0 (no variation) | 76.9 (210) | 1 (adequate) | 81.7 (223) |  |
| 1 (variation) | 23.1 (63) | 2 (not adequate) | 18.3 (50) |  |
| **Breastfeeding type** |  | **Frequency of toothbrushing** |  |  |
| 0 (not breastfed) | 17.2 (47) | 1 (once a day) | 30.4 (83) |  |
| 1 (exclusive breastfed) | 59.4 (162) | 2 (twice a day) | 58.2 (159) |  |
| 2 (breast and bottled fed) | 23.4 (64) | 3 (more than twice a day) | 11.4 (31) |  |
| **Consumption of sugared beverages** |  | **Consumption of vegetables/fruits** |  |  |
| 1 (daily) | 28.2 (77) | 1 (daily) | 41.8 (114) |  |
| 2 (weekly) | 56.4 (154) | 2 (weekly) | 53.1 (145) |  |
| 3 (occasionally) | 15.4 (42) | 3 (occasionally) | 5.1 (14) |  |
| **Breastfeeding time** |  | **Use of Pacifier** |  |  |
| 0 (0 months) | 17.2 (47) | 0 (0 months) | 35.2 (96) |  |
| 1 (1-6 months) | 27.8 (76) | 1 (1-36 months) | 29.3 (80) |  |
| 2 (7-12 months) | 35.9 (98) | 2 (36-48 months) | 23.1 (63) |  |
| 3 (> 12 months) | 19.1 (52) | 3 (> 48 months) | 12.4 (34) |  |

Table 2_bis: p-values of the Fisher’s exact tests for marginal independences for the sample of 273 subjects. P-values smaller than 0.001 suggest the four strongest relations. * Significant (p ≤ 0.050), ** Significant (p ≤ 0.010), *** Significant (p ≤ 0.001). The three two-way marginal dependences statistically different from those in Table 2 have little power and are not retrieved in the full-way analysis (see Tables 5_bis and S5).

|  | Frequency of toothbrushing | Oral hygiene status | Consumption of sugared beverages | Consumption of vegetables/fruits | Breastfeeding time | Use of Pacifier | Caries incidence |
| --- | --- | --- | --- | --- | --- | --- | --- |
| Breastfeeding type | 0.570 | 0.216 | 0.306 | 0.915 | **<0.001***** | 0.081 | 0.052 |
| Frequency of toothbrushing |  | 0.595 | 0.682 | 0.201 | 0.343 | **0.025*** | 0.664 |
| Oral hygiene status |  |  | **<0.001***** | 0.224 | **0.033*** | 0.140 | **<0.001***** |
| Consumption of sugared beverages |  |  |  | 0.698 | 0.215 | 0.333 | **<0.001***** |
| Consumption of vegetables/fruits |  |  |  |  | 0.577 | 0.194 | 0.496 |
| Breastfeeding time |  |  |  |  |  | **0.002**** | **0.024*** |
| Use of Pacifier |  |  |  |  |  |  | 0.572 |

Table 3_bis: p-values of Fisher's exact tests for the most interesting conditional independences given the Oral hygiene status, based on the sample of 273 subjects. Not Significant p-values suggest independence relationships among pair of variables (left) given a specific level of Oral hygiene status. Since for all three pairs of variables the p-values are not significant for both the levels of the oral hygiene, the conclusion is that these pairs of variables are conditional independent.

|  |  | Oral hygiene status | |
| --- | --- | --- | --- |
|  |  | 1 (adequate) | 2 (not adequate) |
| Consumption of sugared beverages | Caries incidence | 0.245 | 1.000 |
| Breastfeeding time | Caries incidence | 0.716 | 0.100 |
| Breastfeeding time | Consumption of sugared beverages | 0.907 | 1.000 |

Table 4_bis: p-values of Fisher's exact tests for the most interesting conditional independence given Breastfeeding time, based on the sample of 273 subjects. Not Significant p-values suggest independence relationships between pair of variables (left) given a specific level of Breastfeeding time. Since for all three pairs of variables the p-values are not significant for all the four levels of the time of the breastfeeding, the conclusion is that these pairs of variables are conditional independent.

|  |  | Breastfeeding time | | | |
| --- | --- | --- | --- | --- | --- |
|  |  | 0  (0 months) | 1  (1-6 months) | 2  (7-12 months) | 3  (>12 months) |
| Oral hygiene status | Use of Pacifier | 0.228 | 0.337 | 0.111 | 0.342 |
| Oral hygiene status | Breastfeeding type | 1.000 | 0.274 | 0.770 | 1.000 |
| Use of Pacifier | Breastfeeding type | 1.000 | 1.000 | 1.000 | 1.000 |

Table S3_bis: odds-ratios of caries incidence with respect to the other seven selected variables for the sample of 273 subjects. Significant p-value associated to an odds-ratio highlights a useful variable in order to predict the variation of the number of caries. 95% confidence intervals for odds-ratios are also calculated.

| **Breastfeeding type** | **odds-ratio** | **left-end confidence interval** | **right-end confidence interval** | **p-value** | |
| --- | --- | --- | --- | --- | --- |
| 1 VS 0 | 3.132 | 1.262 | 9.506 | | 0.024* |
| 2 VS 0 | 2.352 | 0.824 | 7.767 | | 0.128 |
| 2 VS 1 | 0.751 | 0.368 | 1.464 | | 0.413 |
| **Breastfeeding time** |  |  |  | |  |
| 1 VS 0 | 2.420 | 0.879 | 7.826 | | 0.106 |
| 2 VS 0 | 2.432 | 0.918 | 7.677 | | 0.095 |
| 3 VS 0 | 4.836 | 1.738 | 15.833 | | 0.004** |
| 2 VS 1 | 1.005 | 0.491 | 2.082 | | 0.990 |
| 3 VS 1 | 1.998 | 0.917 | 4.402 | | 0.082 |
| 3 VS 2 | 1.989 | 0.948 | 4.172 | | 0.068 |
| **Frequency of toothbrushing** |  |  |  | |  |
| 2 VS 1 | 0.754 | 0.408 | 1.411 | | 0.371 |
| 3 VS 1 | 0.809 | 0.289 | 2.069 | | 0.669 |
| 3 VS 2 | 1.072 | 0.399 | 2.593 | | 0.822 |
| **Oral hygiene status** |  |  |  | |  |
| 2 VS 1 | 731.500 | 143.846 | 13433.030 | | <0.001*** |
| **Consumption of sugared beverages** |  |  |  | |  |
| 2 VS 1 | 0.862 | 0.352 | 2.250 | | 0.751 |
| 3 VS 1 | 353.625 | 63.989 | 6710.357 | | <0.001*** |
| 3 VS 2 | 410.000 | 79.867 | 7551.073 | | <0.001*** |
| **Consumption of vegetables/fruits** |  |  |  | |  |
| 2 VS 1 | 0.958 | 0.533 | 1.734 | | 0.888 |
| 3 VS 1 | 1.880 | 0.538 | 5.954 | | 0.293 |
| 3 VS 2 | 1.962 | 0.569 | 6.105 | | 0.256 |
| **Use of Pacifier** |  |  |  | |  |
| 1 VS 0 | 0.839 | 0.419 | 1.656 | | 0.614 |
| 2 VS 0 | 0.570 | 0.250 | 1.231 | | 0.163 |
| 3 VS 0 | 0.698 | 0.255 | 1.729 | | 0.456 |
| 2 VS 1 | 0.679 | 0.289 | 1.537 | | 0.361 |
| 3 VS 1 | 0.832 | 0.296 | 2.144 | | 0.713 |
| 3 VS 2 | 1.226 | 0.410 | 3.481 | | 0.706 |

Table S4_bis: relative/percentage distribution of joint distribution of Breastfeeding type and time for the sample of 273 subjects. It highlights structural zeros.

|  |  | | Breastfeeding type | | |
| --- | --- | --- | --- | --- | --- |
|  |  |  | 0 | 1 | 2 |
|  | Breastfeeding time | 0 | 17.21 | 0.00 | 0.00 |
|  |  | 1 | 0.00 | 21.25 | 6.59 |
|  |  | 2 | 0.00 | 24.91 | 10.99 |
|  |  | 3 | 0.00 | 13.19 | 5.86 |

Table 5_bis: F-tests for the estimated UGM considering the sample by of 273 subjects. Each row corresponds to a test that tests if a single variable or an interaction between pair of variables can be removed from the estimated UGM without losing information. Significant p-values suggest that the preferable model is the UGM.

|  | Df | Deviance | AIC | F-value | P-value |
| --- | --- | --- | --- | --- | --- |
| UGM | - | 144.16 | 450.96 | - | - |
| Oral hygiene status | 1 | 284.24 | 589.04 | 527 | <0.001*** |
| Consumption of sugared beverages | 2 | 348.00 | 650.80 | 383 | <0.001*** |
| Breastfeeding time | 3 | 194.52 | 495.32 | 63 | <0.001*** |
| Breastfeeding type | 2 | 247.43 | 550.23 | 194 | <0.001*** |
| Use of pacifier | 3 | 145.44 | 446.24 | 2 | 0.186 |
| Caries incidence | 1 | 348.69 | 653.49 | 769 | <0.001*** |
| Oral hygiene status - Consumption of sugared beverages | 2 | 336.50 | 639.30 | 362 | <0.001*** |
| Oral hygiene status - Caries incidence | 1 | 324.70 | 629.50 | 679 | <0.001*** |
| Oral hygiene status - Breastfeeding time | 3 | 153.02 | 453.82 | 11 | <0.001*** |
| Breastfeeding time - Breastfeeding type | 6 | 396.17 | 690.97 | 158 | <0.001*** |
| Breastfeeding time - Use of pacifier | 9 | 170.95 | 459.75 | 11 | <0.001*** |
