# Supplementary material for: Probabilistic graphical modelling of early childhood caries development
Source: PLoS One. 2023 Oct 30;18(10):e0293221. doi: 10.1371/journal.pone.0293221 (PMC10615302; doi:10.1371/journal.pone.0293221)
Supplement: Attachment — Submitted filename: Referee2_fn.docx [file pone.0293221.s003.docx]

**Answers to the Referee Report 2 on the paper:**

**Probabilistic graphical modelling of early childhood caries development (PONE-D-22-04095)**

First of all, we would like to thank the Referee for his/her careful review of the paper. We did our best to revise the article according to his/her valuable suggestions, for which we are grateful. Please find below our answers (in blue) to the comments raised by the Referee. All the changes in the manuscript are in red. Please note that we added an author who recently started to collaborate with us and whose collaboration was instrumental to the production of this revision.

The article is well-written. The statistical analysis of the paper, which is mainly based on undirected graphical models, is well done and described. However, as a major comment, some of the considered variables are, in principle, of numerical type. Examples are: “Use of pacifier”, “Breastfeeding time”, and “Frequency of toothbrushing”. So, transforming them as nominal variables amounts to a twofold loss of information: 1) from numerical to ordinal, and 2) from ordinal to nominal. If these variables had been left in their original nature, the authors could have found an appropriate statistical model that would allow them to take advantage of this additional information. Authors need to discuss this aspect to convince the reader about their choice.

You are absolutely right: transforming numerical variables in categoric ones causes a loss of information. However, this conversion is often done in order in order to have a more manageable dataset and to make more convenient the model. In our case, we could obtain a more direct and a more understandable interpretation of the results. We want to highlight that in such transforming process, we followed the guidelines of experts for the choices of the levels of the variables at stake. Furthermore, we also verified the robustness of our main results to different, reasonable discretizations of the numerical variables into the categorical ones.

Section 3.1: replace “273 and 234 observations identifies the same risk” with “273 and 234 observations identify the same risk”

The sentence has been modified as suggested.

Section 3.1: replace “(see also the section Model Verification)” with “(see also Section 3.3)”

The sentence has been modified as suggested.

Conclusions: replace “with a more effective and efficient prevention strategies” with “with more effective and efficient prevention strategies”

The sentence has been corrected.

Figure 1: replace “34” with “3<4”.< p=""><4”.<>

The errors in Figure 1 have been corrected.
