# Supplementary material for: Probabilistic graphical modelling of early childhood caries development
Source: PLoS One. 2023 Oct 30;18(10):e0293221. doi: 10.1371/journal.pone.0293221 (PMC10615302; doi:10.1371/journal.pone.0293221)
Supplement: Attachment — Submitted filename: Response_to_reviwers.docx [file pone.0293221.s004.docx]

PONE-D-22-04095R1

Probabilistic graphical modelling of early childhood caries development

PLOS ONE

1. In the response to Reviewer #1, the authors say they are using listwise deletion of missing data for simplicity but in the paper they mention that UGMs can accommodate missing data but do not mention in the discussion how this could be accomplished in UGMs. What missing data assumptions (eg assuming data are Missing Completely at Random or data are Missing at Random) do UGMs make? In addition, the paper would be much more useful if the authors – perhaps in the Discussion section – explain how this UGM can be expanded to accommodate missing data.

Thanks for the comment. The MAR property is a key condition for the validity of inference based on the likelihood. When it does not hold, estimates will be biased. The MAR assumption is difficult to verify in practice and its plausibility relies on substantive reasonableness. However, as demonstrated in [Edwards, 2000], "It is possible to evaluate the plausibility of the MAR property, even without detailed knowledge of the missing data process.”

As suggested, in the Discussion section, a sentence about the issue of missing data has been added in the manuscript.

2. In the response to Reviewer #1, the authors note their preference to report results with N=234 after dropping the 39 children from 12 ethnicities. Although the authors have clearly stated who is excluded in the n=39, they have not clearly defined who is included in the N=234. Clearly state who the non-”ethnic” group of 234 includes; is it Italian-born children, children of Italian-born parents, children of Southern European parents, something else?

The considered N=234 children are Italian-born ones, with Italian-born parents. As suggested, a sentence about this point has been added in the Data preparation section.

3. The revised paper made no changes to rely less on p-values as Reviewer #1 requested – Tables 2,3,4 only present p-values with no measures of strength of association such as odds ratios (and 95% confidence intervals) or correlations which would convey the information much better to readers. Adding some measure of strength of association (ORs or correlations) as labels on the edges of the graph in Figure 2 would be very helpful with understanding and interpreting the results.

Thank you for your comment. We performed the Chi-squared independence test for all the pairs of variables that our model assesses as dependent (that is, linked by an edge), by considering the corresponding contingency tables.

Here is the procedure for the variables “Caries Incidence” and “Oral Hygiene Status”:

The contingency table is:

|  | Oral Hygiene Status | |
| --- | --- | --- |
| Caries Incidence | 1 | 2 |
| 0 | 187 | 1 |
| 1 | 10 | 36 |

And the value of the Test Statistic Chi-squared =161.95 (with df=1),

and p-value<2.2 e-16

The results for all the other pairs are included in the following table, and they confirm the results of our model: all these variable pairs can be considered as dependent.

| Variable1 | Variable2 | Test Stat. Chi-squared value | df | p-value |
| --- | --- | --- | --- | --- |
| Oral Hygiene Status | Consump. of Sugar Beverages | 190.45 | 2 | <2.2 e-16 |
| Oral Hygiene Status | Breastfeeding Time | 8.3226 | 3 | 0.03979 |
| Breastfeeding Type | Breastfeeding Time | 236.48 | 6 | <2.2 e-16 |
| Use of pacifier | Breastfeeding Time | 22.686 | 9 | 0.006941 |

Nevertheless, we decided not to include these results in the paper since they can be misunderstood since they are related to tests of independence, while our model is a conditional dependency one. Moreover, in Probabilistic Graphical Models, it is used to compare different models by using the likelihood function, but this is not the case in our study. For these reasons, we finally decided to leave the Tables with the p-values, as we believe that the interest readers can be familiar with them.

4. Strongly suggest removing p-values in the text corresponding to kappas which only test that kappas differ from 0; even kappas below researchers’ thresholds for adequate agreement almost always significantly exceed 0, so the p-values are not useful.

As suggested, the p-values related to kappas have been removed from the	manuscript.

5. In the response to Reviewer #1, the authors wrote “As proposed, in Table 1 the counts have been added and the percentages rounded.” But Table 1 has not been modified in the revision.

We are sorry, but in the submission process of the revised manuscript, we lost the new version of Table 1. Now the correct version of Table 1 has been added.

6. Reviewer #2 identified a “major comment” about impacts of reducing scale from numeric to ordinal to dichotomous. The response explains this was done for convenience to obtain a manageable dataset. However, they do not address the reviewers’ observation that information loss occurs. This should at least be added to the Discussion.

As suggested, a sentence about the information loss due to the variable trasformation (from ordinal to dichotomous) has been added in the Discussion section.

7. Some typographical errors require attention:
   a. “A kappa values for…” should be “Kappa values for…”
   b. Further edit the paragraph to refer to incidence instead of variation. Change text from
   “…zero if there is no variation…”
   to
   “…zero if there is no increment or negative increment...”
   c. In Figure 2, change from “CARIES VARIATION” to “CARIES INCIDENCE”

As suggested, we revised all the manuscript.

We fixed the aforementioned typographical errors and other ones we found in the revision process.

As suggested, the label in Figure 2 has been changed.
